# Comparative Shotgun Proteomics Reveals the Characteristic Protein Signature of Osteosarcoma Subtypes

**DOI:** 10.3390/cells12172179

**Published:** 2023-08-30

**Authors:** Maram Alaa, Nouran Al-Shehaby, Ali Mostafa Anwar, Nesma Farid, Mustafa Shaban Shawky, Manal Zamzam, Iman Zaky, Ahmed Elghounimy, Shahenda El-Naggar, Sameh Magdeldin

**Affiliations:** 1 Immunology and Microbiology Research Program, Basic Research Unit, Research Department, Children’s Cancer Hospital Egypt 57357, Cairo 11441, Egypt; 2 Tumor Biology Research Program, Basic Research Unit, Research Department, Children’s Cancer Hospital Egypt 57357, Cairo 11441, Egypt; 3 Proteomics and Metabolomics Research Program, Basic Research Unit, Research Department, Children’s Cancer Hospital Egypt 57357, Cairo 11441, Egypt; 4 Clinical Research Unit, Research Department, Children’s Cancer Hospital Egypt 57357, Cairo 11441, Egypt; 5 Pathology Department, Children’s Cancer Hospital Egypt 57357, Cairo 11441, Egypt; 6 Pediatric Oncology Department, Children’s Cancer Hospital Egypt 57357, Cairo 11441, Egypt; 7 Pediatric Oncology Department, National Cancer Institute, Cairo University, Cairo 12613, Egypt; 8 Radio Diagnosis Department, Children’s Cancer Hospital Egypt 57357, Cairo 11441, Egypt; 9 Radio Diagnosis Department, National Cancer Institute, Cairo University, Cairo 12613, Egypt; 10 Musculoskeletal Tumor Surgery Unit, Children’s Cancer Hospital Egypt 57357, Cairo 11441, Egypt; 11 Department of Orthopedic Surgery, Faculty of Medicine, Cairo University, Cairo 12613, Egypt; 12 Regenerative Medicine Research Program, Basic Research Unit, Research Department, Children’s Cancer Hospital Egypt 57357, Cairo 11441, Egypt; 13 Department of Physiology, Faculty of Veterinary Medicine, Suez Canal University, Ismailia 41522, Egypt

**Keywords:** pediatric cancer, osteosarcoma, proteomics

## Abstract

Osteosarcoma is a primary malignant bone tumor affecting adolescents and young adults. This study aimed to identify proteomic signatures that distinguish between different osteosarcoma subtypes, providing insights into their molecular heterogeneity and potential implications for personalized treatment approaches. Using advanced proteomic techniques, we analyzed FFPE tumor samples from a cohort of pediatric osteosarcoma patients representing four various subtypes. Differential expression analysis revealed a significant proteomic signature that discriminated between these subtypes, highlighting distinct molecular profiles associated with different tumor characteristics. In contrast, clinical determinants did not correlate with the proteome signature of pediatric osteosarcoma. The identified proteomics signature encompassed a diverse array of proteins involved in focal adhesion, ECM-receptor interaction, PI3K-Akt signaling pathways, and proteoglycans in cancer, among the top enriched pathways. These findings underscore the importance of considering the molecular heterogeneity of osteosarcoma during diagnosis or even when developing personalized treatment strategies. By identifying subtype-specific proteomics signatures, clinicians may be able to tailor therapy regimens to individual patients, optimizing treatment efficacy and minimizing adverse effects.

## 1. Introduction

Osteosarcoma is the most prevalent malignant primary bone tumor, constituting almost one-fifth of all primary sarcomas and around 20–22% of all primary malignant bone tumors. Although rare, pediatric osteosarcoma affects approximately 8.7% of children under 20 years old and is more common in males [1,2]. It has a bimodal age distribution, with the first peak in the second decade of life and the second peak in older individuals [3]. The five-year overall survival rate for patients with non-metastatic osteosarcoma is 60%, whereas patients with lung or bone metastasis have 20% and 13% five-year overall survival rates, respectively. Similarly, patients with recurring diseases have a poor prognosis [4].

Osteosarcoma is a mesenchymal cell tumor that is classified according to the World Health Organization (WHO) into conventional (osteoblastic, chondroblastic, and fibroblastic) and non-conventional (telangiectatic, small cell, high-grade surface, and low-grade) [5]. However, it is difficult to determine the biological differences between these subtypes because their identification relies heavily on sampling, and heterogeneous histologies are often present in a single tumor. Therefore, the commonality of many histologies in a single tumor makes it challenging to draw significant conclusions about the differences between these subtypes [6].

The development of osteosarcoma involves complex etiological factors and pathogenetic mechanisms, but significant progress has been made in understanding its causes. Research efforts in recent decades have been focused on identifying “driver” mutations, which are present in cases of inherited predisposition and sporadic osteosarcoma [7]. These mutations are found in cancer-causing genes, also known as driver genes, and give cancer cells a growth advantage, leading to the outgrowth of the tumor clone. Patients with germline disorders, including Li Fraumeni syndrome, Rec Q abnormalities (Werner syndrome, Rothmund–Thompson syndrome, and Bloom syndrome), and inherited retinoblastoma, are more likely to develop osteosarcoma. CDKN2A, PTEN, mTOR, and TGF-Beta are other driver genes implicated in osteosarcoma etiology [8]. Although advances in high throughput genomics and transcriptomics-based screening have increased our understanding of the genetic factors that contribute to osteosarcoma development, the characterization of the disease proteomics landscape is still in its early stages [9].

Proteins are essential for all cellular functions and play a crucial role in disease pathogenesis and progression. Furthermore, proteomic techniques based on mass spectrometry (MS) have emerged as critical tools in biomarker discovery, prognosis, and treatment follow-up. In the current study, we aimed to investigate the proteomic signature of 33 pediatric osteosarcoma subtypes. We further characterized the relationship between clinical variables of patient outcomes and proteome signatures of osteosarcoma subtypes.

## 2. Materials and Methods

### 2.1. Patients Details

Archived formalin-fixed paraffin-embedded (FFPE) osteosarcoma tissue specimens from 33 patients at the Children’s Cancer Hospital Egypt 57,357 (CCHE) between 2007 and 2015 were provided by the pathology department. Samples were obtained after the Institutional Review Board’s (IRB) approval of CCHE’s waiver of consent (7.20.6). All FFPE samples provided in this study were obtained as part of an initial biopsy at the time of diagnosis before induction chemotherapy. Patient information and follow-up data are available in the Supplementary Table S1.

### 2.2. Tissue Sample Processing

Four 10-µm scrolls were combined and subjected to dewaxing followed by direct tissue trypsinization for each sample. Dewaxing was performed through three incubations in 800 μL of xylene for 1 min each, followed by centrifugation. Sections were then rehydrated through a series of ethanol and distilled water washes (100% ethanol for 2 min, 95% ethanol for 1 min, 70% ethanol for 1 min, distilled water for 1 min). The tissue pellets were dried using speed vacuuming for 20 min at room temperature (RT) after rehydration. Samples were then subjected to direct protein digestion. Briefly, dried tissue pellets were, as in [10], re-suspended in 20 ng/μL trypsin in 50 mM ammonium bicarbonate at pH 8.0, where the volume was adjusted according to the size of the sections (1 μL/mm^2^). Samples were incubated overnight at 37 °C, after which trypsinization was deactivated by adding 1–2 μL of 50% trifluoroethanol (TFA) per sample and centrifuged at 3000 rpm for 5 min, then incubated at RT for 1 min. The resultant peptide mixture was desalted using C18 stage tips (Pierce™ C18 Spin Tips; prod#84850) and sonicated for 15 min prior to LC–MS/MS injection. Peptides were quantified using the BCA quantification assay. 

### 2.3. LC–MS/MS Analysis

Samples were analyzed by nanoLC–MS/MS analysis and carried out using a TripleTOF 5600+ mass spectrometer (AB Sciex, Toronto, ON, Canada) coupled with an Eksigent nanoLC-400 autosampler and an Ekspert nanoLC 425 pump at the front end in Trap and Elute modes. Samples were automatically injected into a trap column on CHROMXP C18-CL 5 um (10 × 0.5 mm) (SCIEX, Canada). Peptides were performed using a 3 µM ChromXP C18CL, 120 A, 150 × 0.3 mm (SCIEX, Canada) reversed-phase column at a 5 µL/min flow rate. A linear gradient of solution A (100% water containing 0.1% formic acid) and B (100% acetonitrile containing 0.1% formic acid) from 3 to 80% B over 55 min. Calibration was scheduled during the batch to correct any possible TOF deviation using 50 fmol of PepCalMix (MS synthetic peptide calibration kit, SCIEX, Canada). The top 40 most abundant parent ions with charge states 2–5 were picked for subsequent fragmentation with an exclusion time of 8 ms. Analyst TF1.7.1 (SCIEX, Canada) recorded peptide spectra over the mass range of 400 to 1250 Da for MS1 and 170 to 1500 for MS2. For MS2 fragmentation, collision-induced dissociation (CID) was used. The MS proteomic data were deposited in the PRIDE repository with the study ID number PXD040681.

### 2.4. Clinical Data Analysis

All statistical analyses were performed using R statistical environment 4.1.3 (10 March 2023). Descriptive statistics were summarized for categorical variables as frequencies, while continuous variables were summarized as the mean and the median. For the univariate and multivariate survival analyses, hazard ratios, 95% confidence intervals (95% CI), and log-rank differences between groups were derived using the “survminer” and “survival” packages with the endpoints of overall survival (OS) and event-free survival (EFS). The OS was calculated from the initial diagnosis to the last follow-up or death. EFS was defined as the time interval from the initial diagnosis to the time of an event, namely tumor progression, recurrence, or death. Both OS and EFS were estimated by the Kaplan–Meier method. Two patients who underwent upfront surgery and one who received palliative treatment were excluded from the analysis. Multivariate analysis was performed for statistically significant OS and/or EFS univariate analyses.

### 2.5. Proteomics Identification

Mascot generic format (mgf) files were generated from raw files using a script supplied by AB Sciex. MS/MS spectra of samples were searched using the X Tandem algorithm within SearchGUI (Galaxy version 3.3.12) and Peptide shaker (Galaxy version 1.16.38) against Uniprot Homosapiens (Swiss-Prot and TrEMBL databases containing 195,194 proteins), with target and decoy sequences. The search of all fully and semi-tryptic peptide candidates was adjusted up to 2 missed cleavages maximum. Precursor mass and fragment mass were identified with an initial mass tolerance of 20 ppm and 10 ppm, respectively. Carbamidomethylation of cysteine (+57.02146 amu) was considered a static modification, with oxidation at methionine (+15.995), acetylation of the protein at the N- terminal and K (+42.01 amu), and pyrrolidone from the carbamidomethylated C (−17.03 amu) as variable modification. The false discovery rate (FDR) was set at 1% at the protein level.

### 2.6. Proteomic Data Analysis 

Before the analysis, data normalization was performed using probabilistic quotient normalization (PQN) [11]. Features with more than 50% missing values per group were removed. Then, a modified imputation strategy was employed on the rest of the features. Median random imputation with a range of ±1% around the median was applied. This approach maintains the features’ median in a group and prevents tied observations in the data [12]. Finally, auto-scaling (mean-centered and divided by the standard deviation of each variable) was performed. To detect the statistically significant proteins between the groups, a *t*-test or Wilcoxon Mann–Whitney test (based on the data distribution tested using the Shapiro–Wilks test) was used with a false discovery rate (FDR) of 5%, a *p*-value < 0.05, and a fold change of (FC2) ± 2. For more than two groups, an ANOVA was used with the same parameters. Cluster analysis using features under an FDR of 10% was applied to the data using correlation distance and ward.D clustering 16, represented as a heatmap. Also, multivariate statistical analyses were performed, including principal component analysis (PCA) using in-house R codes, and the ggplot2 package was used for graphical visualization. Pathway enrichment analysis employing the Kyoto Encyclopedia of Genes and Genomes (KEGG) (ref) was performed using the significant features on the g:Profiler web server [13]. Protein–protein interaction (PPI) network construction and GO enrichment analysis were performed using the STRING database [14]. Cytoscape (version 3.9.1) was used to visualize the PPI networks.

## 3. Results

### 3.1. Patient Cohort Description, Clinical Characteristics, and Outcomes

The patient’s clinical characteristics are described in Table 1. Samples were chosen based on the availability of enough tumor tissue for proteomics analysis.

**Figure 1:**
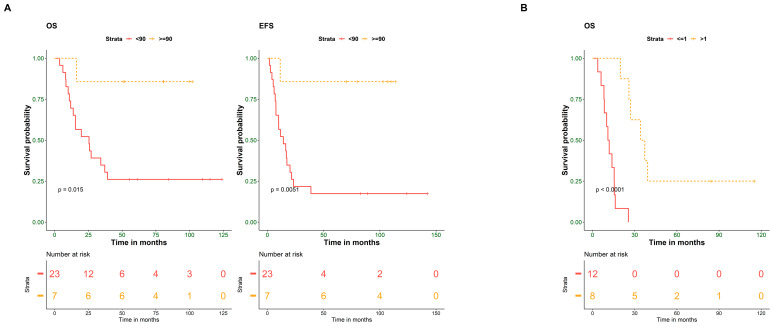
Histological response to chemotherapy and years-to-event predict the outcome of osteosarcoma patients. (**A**) Kaplan–Meier plot of overall (OS) and event-free survival (EFS) for 30 OS patients stratified according to histological response to chemotherapy (≥90% and <90%). (**B**) Kaplan–Meier plot of OS for patients stratified according to years-to-event (≤1 year and >1 year). Survival probability (*y*-axis) and time indicated in months (*x*-axis). *p*-values were calculated using the log-rank test.

In univariate analysis, both histological response to chemotherapy and years-to- event showed significant contributions to the patient’s outcome (OS *p*-value = 0.01, EFS *p*-value = 0.005, and OS *p*-value = 0.00003, respectively) (Figure 1A,B), while pathological subtypes, initial metastasis, and tumor volume did not correlate with the patient’s outcomes (Table 2).

Multivariate analysis was performed on clinical criteria with a *p*-value ≤ 0.1 in the univariate analysis (histological response to chemotherapy, age group, initial metastasis, and years-to-event). Although initial metastasis is a known prognostic factor for osteosarcoma tumors, due to the small sample size, histological response to chemotherapy was the only factor with a significant effect on both OS and EFS of the patients (OS, *p*-value = 0.0345; EFS, *p*-value = 0.0295) (Table 3). 

In addition, a separate multivariate analysis was performed for the 20 patients with an event. An event is described as a recurrence, metastasis, or death. Only years-to-event significantly affected OS (OS, *p*-value = 0.00162), as shown in Table 4.

### 3.2. Clinical Determinants Do Not Correlate with the Proteome Signature of Pediatric Osteosarcoma

**Figure 2:**
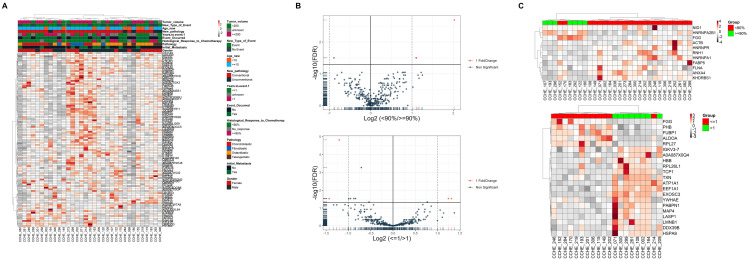
Clinical features do not affect the proteome signature of osteosarcoma patients. (**A**) Heat map and hierarchical clustering based on the total proteome identified. (**B**) Volcano plots of all proteins significantly altered by the (top) histological response to chemotherapy and (bottom) years-to-event (log2-fold-change threshold = 1, Benjamini–Hochberg corrected *p*-value threshold = 0.1). (**C**) Heat map and hierarchical clustering based on the top 20 genes differentiated between groups of (top) Histological response to chemotherapy and (bottom) years-to-event. Heat map colors are based on the *z*-scored (log2) intensity values. Grey and red correspond to decreased and increased expression levels, respectively.

Shotgun proteomics analysis identified 4305 proteins across all 33 samples (Supplementary Table S2). To explore the intrinsic proteome signature, unsupervised hierarchical clustering using the consensus clustering algorithm was used for proteins that appeared in 80% or more of all samples (Figure 2A). The proteome did not identify a clear signature segregating any clinical features. Accordingly, we performed differential protein expression between samples based on clinical characteristics that significantly impact the patient’s outcome. Next, we screened for differentially expressed proteins (DEPs). Only three (KHDRBS1, ANXA4, and HNRNPA2B1) and four proteins (EXOSC3, ALDOA, FUBP1, and HSPA9) were found to be significant (*p*-value ≤ 0.05 and log2 fold change (?)) (Figure 2B) (Table S3) for histological response and years-to-event, respectively. Hierarchical clustering heatmap for the histological response to chemotherapy (≥90% (n = 7) and <90% (n = 23)) as well as years-to-event (>one year (n = 8) and <1 year% (n =13)) using ordered *p*-values for the top 20 genes differentiated between the two groups (Figure 2C). 

However, principal component analysis (PCA) (Figure S1A) did not differentiate between groups within both clinical characteristics. Partial least squares discrimination analysis (PLS-DA) showed segregation in both clinical aspects but with no significance (Figure S1B,C).

### 3.3. Pathological Subtypes Are Defined by Different Proteome Signatures

**Figure 3:**
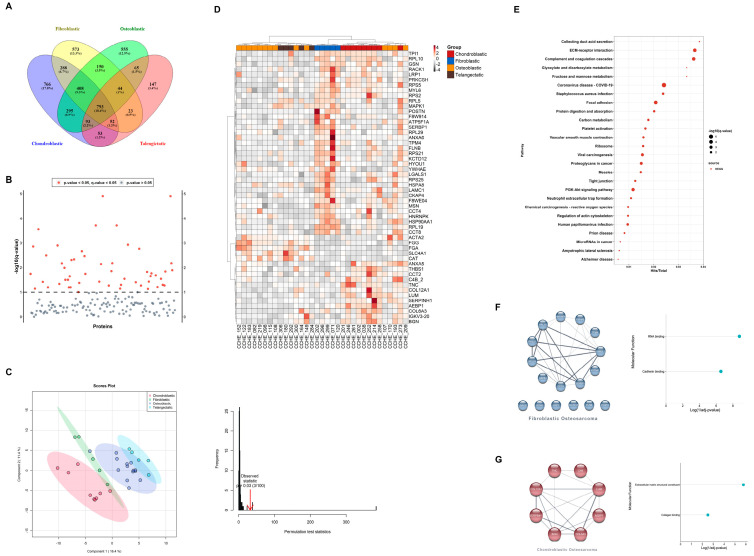
Proteome signatures of osteosarcoma samples segregate patients according to the pathological subtypes. (**A**) Venn diagram showing protein distribution among pathological subtypes. (**B**) Anova diagram showing differentially expressed proteins (DEPs) among pathological subtypes (*p*-value < 0.05). (**C**) Partial least squares discrimination analysis (PLS-DA) based on DEPs showing segregation of pathological subtypes (left) and PLS permutation plot showing PLS-DA significance (right). (**D**) Heat map and hierarchical clustering based on DEPs differentiated between pathological subtypes. Heat map colors are based on the *z*-scored (log2) intensity values. Grey and red correspond to decreased and increased expression levels, respectively. (**E**) KEGG pathways according to DEPs among pathological subtypes. (**F**) PPI networks and molecular functions of DEPs unique to fibroblastic osteosarcoma. (**G**) PPI networks and molecular functions of DEPs unique to chondroblastic osteosarcoma, respectively.

Osteosarcoma pathological subtypes show controversial clinical significance in the literature [15], since they often present with mixed histologies. Therefore, we extended our analysis to compare proteome signatures among pathological subtypes to evaluate whether the difference in subtypes shows up in the proteome signatures. Among pathological subtypes, 793 proteins (18.4%) were shared by all subtypes, whereas 408 proteins (9.5%) were shared among the conventional subtypes (chondroblastic, osteoblastic, and fibroblastic). A total of 573, 555, 766, and 147 proteins were unique to fibroblastic, osteoblastic, chondroblastic, and telangiectatic subtypes, respectively (Figure 3A). Differential expression analysis between pathological subtypes identified 39 significant proteins (Figure 3B, Table S4). 

PCA analysis showed mild segregation of chondroblastic and fibroblastic groups, while osteoblastic and telangiectatic groups showed an overlapped signature (Figure S1D). Using PLS-DA based on the protein expression of DEPs, a separation between pathological subtypes was observed (Figure 3C), where chondroblastic and fibroblastic groups showed better segregation than that observed with osteoblastic and telangiectatic groups. A hierarchical clustering heat map using DEPs showed a similar pattern of sample segregation based on pathology (Figure 3D). 

Pathway analysis of the 39 DEPs using KEGG revealed focal adhesion (q-value = 0.0001), ECM-receptor interaction (q-value = 0.0002), PI3K-Akt signaling pathways (q-value = 0.0005), and proteoglycans in cancer (q-value = 0.0017) among the top enriched pathways (Figure 3E, Table S5).

To determine the relationships among DEPs, PPI networks were built to include seventeen DEPs unique to fibroblastic and eight DEPs unique to chondroblastic (Figure 3F). GO analysis for these sets of DEPs showed enrichment in the RNA-binding and cadherin-binding molecular functions in the fibroblastic subtype. In contrast, in the case of the chondroblastic subtype, enriched molecular functions included extracellular matrix structural constituents and collagen binding (Figure 3F, Table S6).

## 4. Discussion

High-grade osteosarcoma is considered the most common primary malignancy of bones, with a high mortality rate among children and adolescents. Many inherited germline mutations predispose to osteosarcoma; however, most cases are sporadic. Although genome-based studies have provided significant insights into the mutation landscape of osteosarcoma, incorporating proteomics can aid in understanding the complexity of genomic signatures [16]. Thus, this study aimed to use MS methodology to capture the proteomic signature of pediatric osteosarcoma. 

The proteome of 33 pediatric osteosarcoma patients was analyzed and correlated with clinical characteristics. There was no clear segregation based on unsupervised clustering analysis that correlated with clinical characteristics. Furthermore, clinical features impacting patient outcomes in our cohort, histological response, and years-to-event only identified three and four proteins, respectively, which did allow for pathway enrichment analysis. Interestingly, three proteins, KHDRBS1, FUBP1, and HNRNPA2B1, are involved in either RNA processing or RNA modification, highlighting the potential role of RNA regulation in osteosarcoma pathogenesis [17,18,19]. Moreover, HNRNPA2B, an N6-methyladenosine (m6A) reader, has previously been identified as an independent risk factor in osteosarcoma [20,21,22,23]. The aberrant accumulation of m6A modification is the most prevalent modification in eukaryotic RNAs and has been linked to many cancers, including osteosarcoma [24]. Furthermore, genes regulating m6A-mediated gene expression are a promising therapeutic target in osteosarcoma [25,26].

In terms of pathological subtypes, proteomics analysis showed mild segregation with distinct proteome profiles for chondroblastic and fibroblastic subtypes, which correlates with our cohort, where the chondroblastic group was known to be associated with inferior prognosis and response to chemotherapy and shorter overall survival when compared to other groups. Osteoblastic and telangiectatic groups showed overlapping protein signatures, which closely correlated to the histopathological features, as it is known that telangiectatic osteosarcoma is quite similar to osteoblastic osteosarcoma and known to be derived from osteoblasts or stem cells [27,28].

Among the top DEPs identifying fibroblastic groups from other pathological subtypes are POSTN, TPM4, RTN4, HNRNPK, and RACK1, which were directly associated with osteosarcoma progression and prognosis. POSTN, periostin, originally named osteoblast-specific factor 2 (OSF-2), has been involved in regulating the adhesion and differentiation of osteoblasts. It was found that POSTN may have an essential role in tumor progression and may be used as a prognostic biomarker for patients with osteosarcoma [29]. TPM4, or Tropomyosin α-4 chain, was found to be among the differentially expressed proteins in osteosarcoma tissues compared with soft callus tissues [30]. Disrupting RTN4 (reticulon 4)-mediated ER remodeling may impair cancer pathogenicity in U2OS osteosarcoma cells by altering ER homeostasis and nuclear envelope assembly and disassembly during mitosis [31]. Arginine methylation of HNRNPK was shown to suppress the apoptosis of U2OS osteosarcoma cells by interfering with the DDX3-hnRNPK interaction.

On the other hand, the DDX3-hnRNPK interaction with a proapoptotic role may serve as a target for promoting apoptosis in osteosarcoma cells [32]. RACK1 (receptor for activated C-kinase 1) was dose-dependently decreased by catalpol both in MG63 and U2OS osteosarcoma cells, indicating that catalpol could inhibit osteosarcoma progression via epithelial–mesenchymal transition inhibition [33]. GO analysis of DEPs pertaining to fibroblastic osteosarcoma showed enrichment in RNA binding and cadherin binding functions that have been widely shown to have a role in osteosarcoma [34,35,36,37,38].

TNC, LUM, COL12A1, COL6A3, and BGN are among the DEPs that differentiate the chondroblastic group from other subtypes of osteosarcoma. TNC, or tenascin, was highly expressed in osteosarcoma tissues compared to normal [39]. LUM, or lumican, may be positively correlated with the differentiation and negatively correlated with the progression of osteosarcoma [40]. Lumican was also found to be an endogenous inhibitor of TGF-β2 activity, resulting in downstream effector modulation, including pSmad 2, integrin β1, and pFAK, to regulate osteosarcoma adhesion [41]. COL12A1 and COL6A3 were among the proteins enriching the extracellular matrix (ECM) structural constituents and collagen-containing extracellular matrix GO terms. Collagen dysregulation may affect the formation of primary osteosarcoma tumors and metastasis to the lungs [42].

Moreover, upon inhibition of COL6A3, the expression of the PI3K/AKT pathway-related markers changed significantly, suggesting a crucial role for COL6A3 in modulating various aspects of the progression of osteosarcoma, which would provide a potentially effective treatment for osteosarcoma [43]. Transcription of the BGN, biglycan, promoter in bone cells was found to be increased due to elevated levels of intracellular cAMP, which in turn implicates cAMP/protein kinase-A signal transduction pathway in the regulation of biglycan gene expression in osteosarcoma [44]. GO analysis of DEPs defining chondroblastic osteosarcoma revealed dysregulation in the ECM. Previous studies showed that the various components of the ECM, including collagens, fibronectin, laminins, and proteoglycans, may contribute to osteosarcoma progression and metastasis through distinct and intertwining mechanisms, making them potential clinical biomarkers and therapeutic targets. 

Among the DEPs identifying oseoblastic and telangiectatic osteosarcoma subtypes are CAT, FGA, SLC4A1, and ACTA2. In MG63 osteosarcoma cells, HIF-1α was found to inhibit reactive oxygen species accumulation by directly regulating FoxO1, which interfered with CAT catalase activity, thus resulting in anti-oxidation effects [45]. FGA, or fibrinogen alpha chain, was highly elevated in untreated osteosarcoma cells [30]. SLC4A1, solute carrier family 4 member 1, was found to be among the highly significant differentially expressed genes in osteosarcoma [46]. ACTA2, or actin alpha 2, was found to be a potential prognostic indicator for osteosarcoma [47].

Although the prognostic impact of known histologic subtypes of high-grade osteosarcoma is not evident in the literature, our study provides a new insight upon which a proteome profile can be used in future larger studies to provide a more prognostically relevant classification of these variants [48]. Therefore, improving diagnostic strategies for early tumor detection may lead to an increase in patient survival [5].

The first limitation of this study that might affect our analysis was the extraction of proteins from FFPE blocks. Additionally, the FFPE preservation method might impact the stability of mitochondrial-related proteins [49]. Moreover, the low number of used samples (the total number was 33) and the lack of normal adjacent tissue are also limitations. Hence, we recommend further analysis of this data, but in larger cohorts using fresh tissues [50].

In conclusion, proteomic analysis of osteosarcoma tissues gave insight into the molecular mechanisms underlying the pathogenesis of osteosarcoma, thus identifying potential biomarkers and therapeutic targets.

## Figures and Tables

**Table 1:** Clinical characteristics of the osteosarcoma cohort.

Variable	No. of Cases
**Age**	**33**
Mean =12.85	
Median =13.32	
**Age group**	
<10	9
≥10	24
**Gender**	**33**
Female	17
Male	16
**Pathology**	**33**
Conventional Subgroup	28
Chondroblastic	9/28
Fibroblastic	5/28
Osteoblastic	14/28
Non-conventional Subgroup	5
Telangectatic	5/5
**Histological response to chemotherapy**	**30**
Good (≥90% necrosis)	7
Bad (<90% necrosis)	23
**Initial metastasis**	**33**
Yes	10
No	23
**Type of event**	**33**
Event	21
>1 year-to-event	8/21
≤1 year-to-event	13/21
No event	12
**Site of event**	**21**
Local recurrence	1
Local recurrence and Distant Metastasis	4
Distant Metastasis	16
Relative tumor size *	**31**
Mean = 629.2	
Median = 268.9	
**Tumor volume**	**31**
≥200	19
<200	12

* *p* < 0.01.

**Table 2:** Univariate analysis of 30 osteosarcoma patients.

Variable	Number of Cases	OS			EFS		
		HR	95% CI	*p*-Value	HR	95% CI	*p*-Value
**Pathology**	30			0.5			0.7
Conventional vs. Non-conventional	25 vs. 5	1.741	0.1319–2.502	0.46	0.8034	0.2342–2.756	0.728
**Conventional**	28			0.8			
Fibroblastic vs. Chondroblastic	4 vs. 8	1.6688	0.3223–8.641	0.542	0.8729	0.2074–3.674	0.853
Fibroblastic vs. Osteoblastic	4 vs. 13	1.7914	0.3843–8.350	0.458	1.0878	0.2926–4.044	0.900
**Histological response to chemotherapy**	30			0.01 *			0.005 *
Good (≥90% necrosis) vs. Bad (<90% necrosis)	7 vs. 23	0.121	0.01602–0.9132	0.0406 *	0.9656	0.01282–0.7273	0.0233 *
**Age group**	30			0.06			0.09
<10 vs. ≥10	8 vs. 22	0.4068	0.1523–1.087	0.0729	0.454	0.1784–1.156	0.0976
**Gender**	30			0.4			0.3
Female vs. Male	15 vs. 15	1.48	0.5825–3.759	0.41	1.563	0.6452–3.778	0.322
**Initial metastasis**	30			0.2			0.09
Yes vs. No	9 vs. 21	1.795	0.6947–4.636	0.227	2.177	0.877–5.402	0.0936
**Tumor Volume**	28			0.9			0.6
≥200 vs. <200	18 vs. 10	1.038	0.3763–2.866	0.942	0.7848	0.4774–3.401	0.629
**Years-to-event**	20			0.00003 ****	Not Applicable		
>1 vs. ≤1	8 vs. 12	0.0365	0.00445–0.2993	0.00205 ****			

* p < 0.01, **** p < 0.001.

**Table 3:** Multivariate analysis of 30 osteosarcoma patients.

Variable	OS			EFS		
	HR	95% CI	*p*-Value	HR	95% CI	*p*-Value
**Histological response to chemotherapy**	0.1137	0.0150–0.8618	0.0354 *	0.1051	0.01382–0.7989	0.0295 *
**Age group**	0.9066	0.8014–1.0256	0.1192	0.9046	0.79947–1.0236	0.1118
**Initial metastasis**	Not Applicable			1.8208	0.70743–4.6862	0.2141

* *p* < 0.01.

**Table 4:** Multivariate analysis of 20 osteosarcoma patients showing events.

Variable	Number of Cases	OS		
		HR	95% CI	*p*-Value
**Histological response to chemotherapy**	20	0.49406	0.056282–4.337	0.52466
**Age group**	20	1.04510	0.908952–1.202	0.53564
**Years-to-event**	20	0.02558	0.002617–0.250	0.00162 **

** p < 0.05.

## Data Availability

The MS proteomic data were deposited to the MASSIVE repository with the study ID number PXD040681. Additional information is available upon request.

## Supplementary Materials

The following supporting information can be downloaded at: https://www.mdpi.com/article/10.3390/cells12172179/s1, Figure S1: Clinical features: proteome signature PCA and PLS-DA; Table S1: Patient information and follow-up data; Table S2: Proteins identified by shotgun proteomics analysis; Table S3: Differentially expressed proteins identified upon comparing proteomes significantly affecting clinical features; Table S4: Differentially expressed proteins between pathological subtypes; Table S5: Pathway analysis of DEPs between pathological subtypes; Table S6: GO analysis for DEPs between pathological subtypes.

## References

[1] Ottaviani G., Jaffe N. (2009). The Epidemiology of Osteosarcoma. Cancer Treat Res..

[2] Corre I., Verrecchia F., Crenn V., Redini F., Trichet V. (2020). The Osteosarcoma Microenvironment: A Complex but Targetable Ecosystem. Cells.

[3] Isakoff M.S., Bielack S.S., Meltzer P., Gorlick R. (2015). Osteosarcoma: Current Treatment and a Collaborative Pathway to Success. J. Clin. Oncol..

[4] Harris M.A., Hawkins C.J. (2022). Recent and Ongoing Research into Metastatic Osteosarcoma Treatments. Int. J. Mol. Sci..

[5] Wadhwa N. (2014). Osteosarcoma: Diagnostic dilemmas in histopathology and prognostic factors. Indian J. Orthop..

[6] Yoshida A. (2021). Osteosarcoma: Old and New Challenges. Surg. Pathol. Clin..

[7] Morrow J.J., Khanna C. (2015). Osteosarcoma Genetics and Epigenetics: Emerging Cancer Treat ResBiology and Candidate Therapies. Crit. Rev. Oncog..

[8] Hameed M., Mandelker D. (2018). Tumor Syndromes Predisposing to Osteosarcoma. Adv. Anat. Pathol..

[9] Bernardini G., Laschi M., Geminiani M., Santucci A. (2014). Proteomics of osteosarcoma. Expert Rev. Proteom..

[10] Matos L.L., Trufelli D.C., de Matos M.G.L., da Silva Pinhal M.A. (2010). Immunohistochemistry as an important tool in biomarkers detection and clinical practice. Biomark Insights.

[11] Dieterle F., Ross A., Schlotterbeck G., Senn H. (2006). Probabilistic Quotient Normalization as Robust Method to Account for Dilution of Complex Biological Mixtures. Application in ^1^H NMR Metabonomics. Anal. Chem..

[12] Overmyer K.A., Shishkova E., Miller I.J., Balnis J., Bernstein M.N., Peters-Clarke T.M., Meyer J.G., Quan Q., Muehlbauer L.K., Trujillo E.A. (2020). Large-Scale Multi-omic Analysis of COVID-19 Severity. Cell Syst..

[13] Raudvere U., Kolberg L., Kuzmin I., Arak T., Adler P., Peterson H., Vilo J. (2019). g:Profiler: A web server for functional enrichment analysis and conversions of gene lists (2019 update). Nucleic Acids Res..

[14] Szklarczyk D., Franceschini A., Wyder S., Forslund K., Heller D., Huerta-Cepas J., Simonovic M., Roth A., Santos A., Tsafou K.P. (2015). STRING v10: Protein-Protein Interaction Networks, Integrated Over the Tree of Life. Nucleic Acids Res..

[15] Misaghi A., Goldin A., Awad M., Kulidjian A.A. (2018). Osteosarcoma: A comprehensive review. SICOT-J.

[16] Lindsey B.A., Markel J.E., Kleinerman E.S. (2016). Osteosarcoma Overview. Rheumatol. Ther..

[17] Sumithra B., Saxena U., Das A.B. (2019). A comprehensive study on genome-wide coexpression network of KHDRBS1/Sam68 reveals its cancer and patient-specific association. Sci. Rep..

[18] Elman J.S., Ni T.K., Mengwasser K.E., Jin D., Wronski A., Elledge S.J., Kuperwasser C. (2019). Identification of FUBP1 as a Long Tail Cancer Driver and Widespread Regulator of Tumor Suppressor and Oncogene Alternative Splicing. Cell Rep..

[19] Maroni P., Luzzati A., Perrucchini G., Cannavò L., Bendinelli P. (2020). Leptin, Leptin Receptor, KHDRBS1 (KH RNA Binding Domain Containing, Signal Transduction Associated 1), and Adiponectin in Bone Metastasis from Breast Carcinoma: An Immunohistochemical Study. Biomedicines.

[20] Chen S., Li Y., Zhi S., Ding Z., Wang W., Peng Y., Huang Y., Zheng R., Yu H., Wang J. (2020). WTAP promotes osteosarcoma tumorigenesis by repressing HMBOX1 expression in an m6A-dependent manner. Cell Death Dis..

[21] Liu H., Qin G., Ji Y., Wang X., Bao H., Guan X., Wei A., Cai Z. (2021). Potential role of m6A RNA methylation regulators in osteosarcoma and its clinical prognostic value. J. Orthop. Surg. Res..

[22] Zhang Y., Wang Y., Ying L., Tao S., Shi M., Lin P., Wang Y., Han B. (2021). Regulatory Role of N6-methyladenosine (m6A) Modification in Osteosarcoma. Front. Oncol..

[23] Han J., Kong H., Wang X., Zhang X. (2022). Novel insights into the interaction between N6-methyladenosine methylation and noncoding RNAs in muscu-loskeletal disorders. Cell Prolif..

[24] He L., Li H., Wu A., Peng Y., Shu G., Yin G. (2019). Functions of N6-methyladenosine and its role in cancer. Mol. Cancer.

[25] Chen X., Xu M., Xu X., Zeng K., Liu X., Pan B., Li C., Sun L., Qin J., Xu T. (2020). METTL14-mediated N6-methyladenosine modification of SOX4 mRNA inhibits tumor metastasis in colorectal cancer. Mol. Cancer.

[26] Deng L.-J., Deng W.-Q., Fan S.-R., Chen M.-F., Qi M., Lyu W.-Y., Qi Q., Tiwari A.K., Chen J.-X., Zhang D.-M. (2022). m6A modification: Recent advances, anticancer targeted drug discovery and beyond. Mol. Cancer.

[27] Sangle N.A., Layfield L.J. (2012). Telangiectatic osteosarcoma. Arch. Pathol. Lab. Med..

[28] Metcalf D.J., Nightingale T.D., Zenner H.L., Lui-Roberts W.W., Cutler D.F. (2008). Formation and function of Weibel-Palade bodies. J. Cell Sci..

[29] Hu F., Shang X.-F., Wang W., Jiang W., Fang C., Tan D., Zhou H.-C. (2016). High-level expression of periostin is significantly correlated with tumour angiogenesis and poor prognosis in osteosarcoma. Int. J. Exp. Pathol..

[30] Chaiyawat P., Sungngam P., Teeyakasem P., Sirikaew N., Klangjorhor J., Settakorn J., Diskul-Na-Ayudthaya P., Chokchaichamnankit D., Srisomsap C., Svasti J. (2019). Protein profiling of osteosarcoma tissue and soft callus unveils activation of the unfolded protein response pathway. Int. J. Oncol..

[31] Bateman L.A., Nguyen T.B., Roberts A.M., Miyamoto D.K., Ku W.-M., Huffman T.R., Petri Y., Heslin M.J., Contreras C.M., Skibola C.F. (2017). Chemoproteomics-enabled covalent ligand screen reveals a cysteine hotspot in reticulon 4 that impairs ER morphology and cancer pathogenicity. Chem. Commun..

[32] Chen C.-C., Yang J.-H., Fu S.-L., Lin W.-J., Lin C.-H. (2021). Arginine Methylation of hnRNPK Inhibits the DDX3-hnRNPK Interaction to Play an Anti-Apoptosis Role in Osteosarcoma Cells. Int. J. Mol. Sci..

[33] Wang L., Xue G.-B. (2017). Catalpol suppresses osteosarcoma cell proliferation through blocking epithelial-mesenchymal transition (EMT) and inducing apoptosis. Biochem. Biophys. Res. Commun..

[34] Lei Y., Junxin C., Yongcan H., Xiaoguang L., Binsheng Y. (2020). Role of microRNAs in the crosstalk between osteosarcoma cells and the tumour microenvironment. J. Bone Oncol..

[35] Yang Z., Li X., Yang Y., He Z., Qu X., Zhang Y. (2016). Long noncoding RNAs in the progression, metastasis, and prognosis of osteosarcoma. Cell Death Dis..

[36] Li Z., Li X., Xu D., Chen X., Li S., Zhang L., Chan M.T.V., Wu W.K.K. (2020). An update on the roles of circular RNAs in osteosarcoma. Cell Prolif..

[37] Jolly M.K., Ware K.E., Xu S., Gilja S., Shetler S., Yang Y., Wang X., Austin R.G., Runyambo D., Hish A.J. (2019). E-Cadherin Represses Anchorage-Independent Growth in Sarcomas through Both Signaling and Mechanical Mechanisms. Mol. Cancer Res..

[38] Kashima T., Kawaguchi J., Takeshita S., Kuroda M., Takanashi M., Horiuchi H., Imamura T., Ishikawa Y., Ishida T., Mori S. (1999). Anomalous Cadherin Expression in Osteosarcoma: Possible Relationships to Metastasis and Morphogenesis. Am. J. Pathol..

[39] Xi Y., Liu J., Shen G. (2022). Low expression of IGFBP4 and TAGLN accelerate the poor overall survival of osteosarcoma. Sci. Rep..

[40] Nikitovic D., Berdiaki A., Zafiropoulos A., Katonis P., Tsatsakis A., Karamanos N.K., Tzanakakis G.N. (2007). Lumican expression is positively correlated with the differentiation and negatively with the growth of human osteosarcoma cells. FEBS J..

[41] Nikitovic D., Chalkiadaki G., Berdiaki A., Aggelidakis J., Katonis P., Karamanos N.K., Tzanakakis G.N. (2011). Lumican regulates osteosarcoma cell adhesion by modulating TGFbeta2 activity. Int. J. Biochem. Cell Biol..

[42] Heng L., Jia Z., Bai J., Zhang K., Zhu Y., Ma J., Zhang J., Duan H. (2017). Molecular characterization of metastatic osteosarcoma: Differentially expressed genes, transcription factors and microRNAs. Mol. Med. Rep..

[43] Guo H.-L., Chen G., Song Z.-L., Sun J., Gao X.-H., Han Y.-X. (2020). COL6A3 promotes cellular malignancy of osteosarcoma by activating the PI3K/AKT pathway. Rev. Assoc. Médica Bras..

[44] Ungefroren H., Cikós T., Krull N.B., Kalthoff H. (1997). Biglycan Gene Promoter Activity in Osteosarcoma Cells Is Regulated by Cyclic AMP. Biochem. Biophys. Res. Commun..

[45] Sun W., Wang B., Qu X.-L., Zheng B.-Q., Huang W.-D., Sun Z.-W., Wang C.-M., Chen Y. (2019). Metabolism of Reactive Oxygen Species in Osteosarcoma and Potential Treatment Applications. Cells.

[46] Jia Y., Liu Y., Han Z., Tian R. (2021). Identification of potential gene signatures associated with osteosarcoma by integrated bioinformatics analysis. PeerJ.

[47] Yao F., Zhu Z.F., Wen J., Zhang F.Y., Zhang Z., Zhu L.Q., Su G.H., Yuan Q.W., Zhen Y.F., Wang X.D. (2021). PODN is a prognostic biomarker and correlated with immune infiltrates in osteosarcoma. Cancer Cell Int..

[48] Lilienthal I., Herold N. (2020). Targeting Molecular Mechanisms Underlying Treatment Efficacy and Resistance in Osteosarcoma: A Review of Current and Future Strategies. Int. J. Mol. Sci..

[49] Valdés A., Bitzios A., Kassa E., Shevchenko G., Falk A., Malmström P.-U., Dragomir A., Segersten U., Lind S.B. (2021). Proteomic comparison between different tissue preservation methods for identification of promising biomarkers of urothelial bladder cancer. Sci. Rep..

[50] Davalieva K., Kiprijanovska S., Dimovski A., Rosoklija G., Dwork A.J. (2021). Comparative evaluation of two methods for LC-MS/MS proteomic analysis of formalin fixed and paraffin embedded tissues. J. Proteom..

