# Supplementary material for: Comparative Shotgun Proteomics Reveals the Characteristic Protein Signature of Osteosarcoma Subtypes
Source: Cells. 2023 Aug 30;12(17):2179. doi: 10.3390/cells12172179 (PMC10487120; doi:10.3390/cells12172179)
Supplement: Supplementary file 1 [file cells-12-02179-s001.zip › Supplementary Figure S1.pdf]

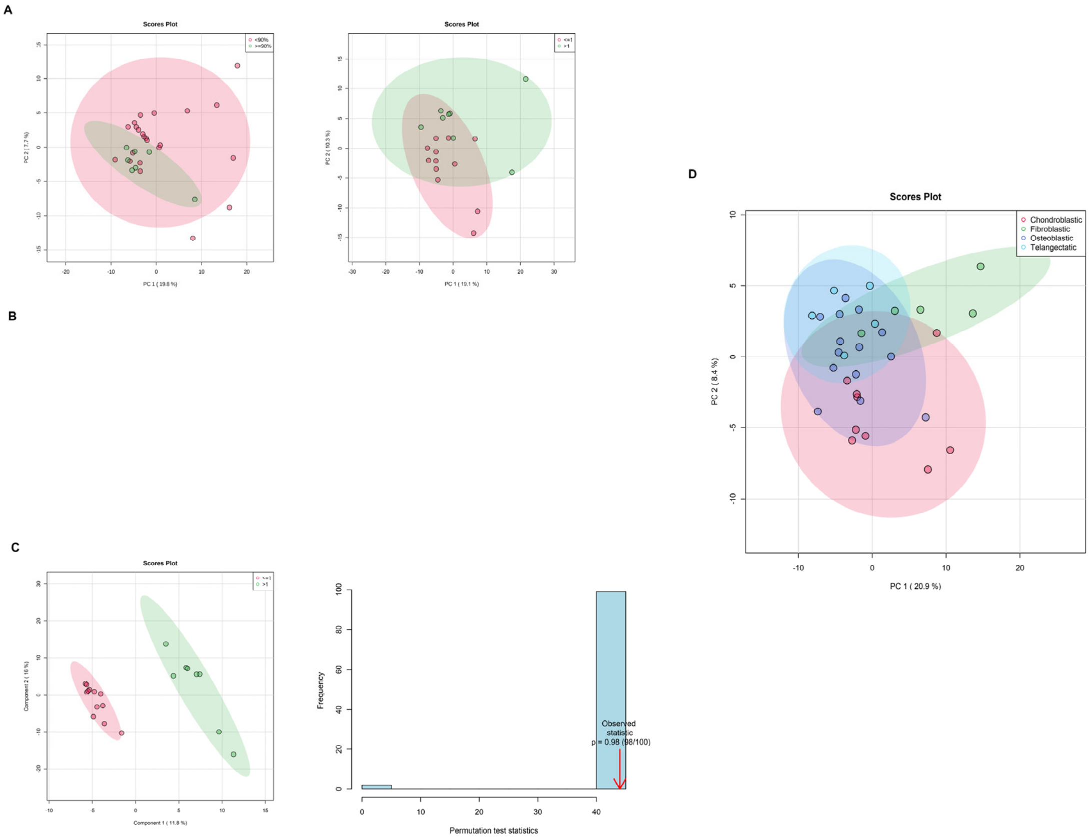

Supplementary Figure S1. Proteome signature of osteosarcoma patients. (A) Principal component analysis (PCA) of whole proteome according to (left) histological response to chemotherapy and (right) years to event. (B) Partial least square discrimination analysis (PLS-DA) of whole proteome according to Histological response to chemotherapy (left) and PLS permutation significance plot (right). (C) PLS-DA of whole proteome according to years to event (left) and PLS permutation significance plot (right). (D) PCA) of whole proteome according to pathological subgroups.
